# A Novel Resolution of Diabetes: C-C Chemokine Motif Ligand 4 Is a Common Target in Different Types of Diabetes by Protecting Pancreatic Islet Cell and Modulating Inflammation

**DOI:** 10.3389/fimmu.2021.650626

**Published:** 2021-04-23

**Authors:** Ting-Ting Chang, Liang-Yu Lin, Jaw-Wen Chen

**Affiliations:** ^1^ Department and Institute of Pharmacology, School of Medicine, National Yang-Ming University, Taipei, Taiwan; ^2^ Department and Institute of Pharmacology, National Yang Ming Chiao Tung University, Taipei, Taiwan; ^3^ School of Medicine, National Yang Ming Chiao Tung University, Taipei, Taiwan; ^4^ Division of Endocrinology and Metabolism, Department of Medicine, Taipei Veterans General Hospital, Taipei, Taiwan; ^5^ Healthcare and Services Center, Taipei Veterans General Hospital, Taipei, Taiwan; ^6^ Division of Cardiology, Department of Medicine, Taipei Veterans General Hospital, Taipei, Taiwan; ^7^ Cardiovascular Research Center, National Yang-Ming University, Taipei, Taiwan

**Keywords:** blood sugar, C-C chemokine motif ligand 4, diabetes mellitus, inflammation, insulin resistance, pancreatic islet cells

## Abstract

Systemic inflammation is related to hyperglycemia in diabetes mellitus (DM). C-C chemokine motif ligand (CCL) 4 is upregulated in type 1 & type 2 DM patients. This study aimed to investigate if CCL4 could be a potential target to improve blood sugar control in different experimental DM models. Streptozotocin-induced diabetic mice, *Lepr^db^*/JNarl diabetic mice, and C57BL/6 mice fed a high fat diet were used as the type 1 DM, type 2 DM, and metabolic syndrome model individually. Mice were randomly assigned to receive an anti-CCL4 neutralizing monoclonal antibody. The pancreatic β-cells were treated with streptozotocin for *in vitro* experiments. In streptozotocin-induced diabetic mice, inhibition of CCL4 controlled blood sugar, increased serum insulin levels, increased islet cell proliferation and decreased pancreatic interleukin (IL)-6 expression. In the type 2 diabetes and metabolic syndrome models, CCL4 inhibition retarded the progression of hyperglycemia, reduced serum tumor necrosis factor (TNF)-α and IL-6 levels, and improved insulin resistance *via* reducing the phosphorylation of insulin receptor substrate-1 in skeletal muscle and liver tissues. CCL4 inhibition directly protected pancreatic β-cells from streptozotocin stimulation. Furthermore, CCL4-induced IL-6 and TNF-α expressions could be abolished by siRNA of CCR2/CCR5. In summary, direct inhibition of CCL4 protected pancreatic islet cells, improved insulin resistance and retarded the progression of hyperglycemia in different experimental models, suggesting the critical role of CCL4-related inflammation in the progression of DM. Future experiments may investigate if CCL4 could be a potential target for blood sugar control in clinical DM.

## Introduction

Systemic inflammation has been related to the progression of hyperglycemia and is suggested to be a potential therapeutic targets in clinical diabetes mellitus (DM) (1, 2). Given that both macro- and microvasculopathy are observed during the early progression of hyperglycemia, systemic inflammation is proposed to be a common background for the deterioration of blood sugar levels and the development of diabetic vasculopathy in clinical DM (3–5). However, systemic inflammation may be modified and varied in different types of DM as well as in metabolic diseases. The detailed inflammatory mechanisms and specified mediators have not been fully clarified.

Inflammatory chemokines are regarded as potential contributors and therapeutic targets in some cardiovascular and metabolic diseases (6). It was shown that C-C chemokine motif ligand (CCL) 4, a chemokine, could be upregulated to modulate the downstream inflammatory cytokines in type 1 DM (7). Circulating CCL4 levels were inversely related to proinsulin levels (8). Furthermore, circulating CCL4 levels are similarly elevated in both type 1 and type 2 DM patients, suggesting the general involvement of CCL4 in different types of DM (9). Recently, CCL4 inhibition was shown to improve ischemia-induced neovasculogenesis in different types of diabetic mice, suggesting the general role of CCL4 in vasculopathy in the presence of hyperglycemia (10). While CCL4 may be related to systemic inflammation and vasculopathy individually in different types of DM (11), the potential role of CCL4 in hyperglycemia has not been clarified.

In this study, both *in vivo* and *in vitro* experiments were conducted to investigate whether directly inhibiting CCL4 could retard the progression of hyperglycemia in different animal models and directly protect pancreatic β-cells. Our findings may hopefully elucidate the particular role of CCL4-related inflammation in the progression of hyperglycemia and provide some novel rationale to the potential strategy targeting on CCL4 to improve blood sugar control in clinical DM.

## Materials And Methods

### *In Vivo* Study

#### Animal Procedures

Six-week-old male BKS.Cg-*Dock7^m^+/+ Lepr^db^*/JNarl (db/db mice) mice, nondiabetic littermate control db/m mice (non-DM mice), C57BL/6 mice, FVB/NJNarl mice, and eight-week-old female NOD/ShiLtJNarl mice were purchased from the National Laboratory Animal Center (Taipei, Taiwan). FVB/NJNarl mice and C57BL/6 mice were acclimated for 2 weeks before being used to generate the type 1 DM and metabolic syndrome models. To generate hyperglycemia in the FVB/NJNarl mice, FVB/NJNarl mice were intraperitoneally injected with streptozotocin (40 mg/kg for 5 days). Blood glucose was evaluated after the mice were fasted for 4 hours. Overall, the mice showed a blood glucose level of at least 250 mg/dL. LabDiet Rodent 5001 [23.9% protein, 5% fat (ether extract), 5% fat (acid hydrolysis); LabDiet, St. Louis, MO, USA] was used as a standard diet. C57BL/6 mice were fed a high fat diet (23.6% protein, 34.9% fat, 25.9% carbohydrates, diet-induced obesity rodent-purified diet with 60% energy from fat-58Y1) for 14 weeks. The male BKS.Cg-*Dock7^m^+/+ Lepr^db^*/JNarl mice and female NOD/ShiLtJNarl mice were not treated with any drug and were allowed to acclimate for 2 weeks before receiving an anti-CCL4 neutralizing monoclonal antibody or isotype control. The female NOD mice were used because a marked gender difference was observed in the incidence of diabetic symptoms (12). The 4~5 mice were housed per cage. Animal experiments were conducted at National Yang-Ming University. Animals were raised according to the regulations of the Animal Care Committee of National Yang-Ming University. All animal-related work was performed under the Institutional Animal Care and Use Committee (IACUC) protocol approved by National Yang-Ming University (IACUC no. 1050909).

Some diabetic mice received an intraperitoneal injection of an anti-CCL4 neutralizing monoclonal rat IgG_2A_ antibody (MAB451, 100 μg; R&D Systems, Minneapolis, MN, USA) 3 times per week for 2 or 4 weeks. In detail, the antibodies were injected after the fasting blood glucose levels were over than 250 mg/dL in STZ-induced type 1 DM model. In metabolic syndrome model, the antibodies were injected after C57BL/6 mice were fed a high fat diet for 14 weeks. In dbdb and NOD mice, the antibodies were injected at the beginning of 8-week-old and 12-week-old, respectively. The antibody was shown to have specific CCL4 lowering effects in STZ-induced diabetic mice model (10). The rat IgG_2A_ isotype (MAB006, 100 μg; R&D Systems) was administered as a control. Body weights and blood sugar concentrations were measured.

### Oral Glucose Tolerance Tests (OGTTs) and Areas Under the Curve (AUCs)

Mice were fasted for 6 hours and orally administered glucose at a dose of 2 g/kg body weight. Blood samples were obtained from tail vein at time 0 (just before glucose load) and at 15, 30, 60, 90 and 120 minutes after glucose administration. In detail, the Abbott Freestyle Optium glucometer was used for the measurements. The blood glucose levels were measured by Abbott Freestyle Optium glucometer strips in 5 seconds. The total and incremental AUCs for plasma glucose during the OGTT were determined by the trapezoidal method (13).

### Homeostasis Model Assessment of Insulin Resistance (HOMA-IR)

Insulin resistance, insulin sensitivity and steady-state β-cell function were determined by the homeostasis model assessment of insulin resistance (HOMA-IR) using fasting glucose and insulin levels. HOMA analysis is an accepted surrogate for measuring insulin resistance in rodents (14). The HOMA-IR index was calculated as [fasting insulin (μU/mL) × fasting glucose (mmol/L)]/22.5 (15).

### Measurement of Insulin Expression in the Pancreas

Histological analyses of insulin expression in the pancreas were performed. The tissues were incubated in a 30% sucrose solution for 24 hours, embedded in OCT compound (Sakura Finetek, Torrance, CA, USA), and frozen in liquid nitrogen. For the insulin expression measurement, the sections were fixed with methanol for 10 minutes, washed briefly with PBS, stained with a monoclonal rat anti-insulin antibody (1:400; Cell Signaling, Beverly, MA, USA) at 37°C for 2 hours, and incubated with an Alexa Fluor 594-conjugated goat anti-rat antibody (Jackson ImmunoResearch, West Grove, PA, USA). Hoechst 33258 (Merck & Co., Rogers, AR, USA), a blue fluorescent dye, was used for the nucleic acid stain. The insulin expressions were observed in six random microscopic fields by a qualitative analysis (n=4 in IgG_2A_-treated group; n=6~13 in the other groups).

### Measurement of Ki-67 and Insulin Expression in the Pancreas

The tissues were fixed in 4% paraformaldehyde and then embedded in paraffin. Sections were deparaffinized and incubated with a rabbit- polyclonal antibody against the murine marker of proliferation Ki-67 (Novus, Centennial, CO, USA) and a guinea pig- polyclonal antibody against murine insulin (GeneTex, Irvine, CA, USA). Antibody distribution was visualized with the EnVision+ Single Reagents/HRP/Rabbit and Liquid DAB+ Substrate Chromogen System (Agilent/Dako, Santa Clara, CA, USA), followed by counterstaining with hematoxylin. The Ki-67- and insulin-positive areas are shown in dark brown. The Ki-67 and insulin expressions were observed in six random microscopic fields by a qualitative analysis (n=4 in IgG_2A_-treated group; n=6~13 in the other groups).

### *In Vitro* Study

#### NIT-1 Cell Culture

The mouse pancreatic β-cell line NIT-1 was purchased from ATCC (no. CRL-2055) and cultured in Ham’s F12K medium supplemented with 10% fetal bovine serum at 37°C in a humidified incubator with an atmosphere of 5% CO_2_. The cell culture medium was exchanged every 48 hours. The NIT-1 cells were harvested and passaged by detaching, aspirating and separating the adherent cells, followed by incubation with cell dissociation buffer (an enzyme-free Hanks’-based solution).

### Cell Proliferation Assay of NIT-1 Cells

Cells were seeded in 96-well plates at a concentration of 1×10^5^ cells per well and preincubated overnight. After preincubation, NIT-1 cells were exposed to STZ (0, 0.75, 1.5, 3, or 6 mM) for 24 hours. The cytotoxicity of STZ on the NIT-1 cells was determined using an MTT assay. Additionally, NIT-1 cells were treated with STZ for 24 hours and then with or without a CCL4 antibody (R&D Systems) at a low dose (0.3 μg/mL) or a high dose (30 μg/mL) for 4 hours. NIT-1 cell proliferation was evaluated using an MTT assay.

### Evaluation of Insulin and CCL4 Concentrations in NIT-1 Cell Supernatants

Equal numbers of NIT-1 cells were seeded in 6-well plates in Ham’s F12K medium supplemented with 5% FBS. NIT-1 cells were treated with STZ for 24 hours and then with or without a CCL4 antibody (R&D Systems) at a low dose (0.3 μg/mL) or a high dose (30 μg/mL) for 4 hours. Supernatant concentrations of insulin and CCL4 released from NIT-1 cells were determined by ELISA (Millipore, Temecula, CA, USA and R&D Systems) according to the manufacturers’ instructions. The ELISA from R&D Systems was designed to measure mouse CCL4 levels in cell culture supernatants, serum, and plasma.

### Evaluation of TNF-α, IL-6, and Insulin Concentrations

Serum protein concentrations of TNF-α and IL-6 were determined by ELISA (R&D Systems) according to the manufacturer’s instructions. Serum concentrations of insulin were determined by ELISA (Millipore) according to the manufacturers’ instructions.

### Transfection of ccr2 and ccr5 siRNA in NIT-1 Cells

NIT-1 cells were transfected with ccr2 and ccr5 siRNA (Santa Cruz Biotechnology, Dallas, TX, USA) using Lipofectamine 2000 (Invitrogen, Carlsbad, CA, USA) in culture medium. Then, cells were treated with CCL4 (1 µg/mL; R&D Systems) for 24 hours.

### Western Blot Analyses

Equal amounts of protein were separated by SDS-PAGE on 4–12% gradient gels under reducing conditions (Bio-Rad Laboratories, Berkeley, CA, USA) and then transferred to nitrocellulose membranes (GE Healthcare, Chicago, IL, USA). The membranes were incubated with antibodies against interleukin (IL)-6 (Santa Cruz Biotechnology), TNF-α, total IRS-1, and p-IRS-1 (Ser-307; Cell Signaling). The expression of IL-6 and TNF-α as determined by immunoblot was normalized to that of β-actin as determined using a mouse monoclonal anti-β-actin antibody.

### Statistics

The results are presented as the means ± standard deviation (SD). Statistical analyses were performed using unpaired Student’s t-test or analysis of variance, followed by Scheffe’s multiple-comparison *post hoc* test. SPSS software (version 14; SPSS, Chicago, IL, USA) was used to analyze the data. A p value <0.05 was considered statistically significant.

## Results

### CCL4 Inhibition Protected Pancreatic Islet Function and Controlled Blood Sugar Levels in Type 1 Diabetic Mice

**Figure 1:**
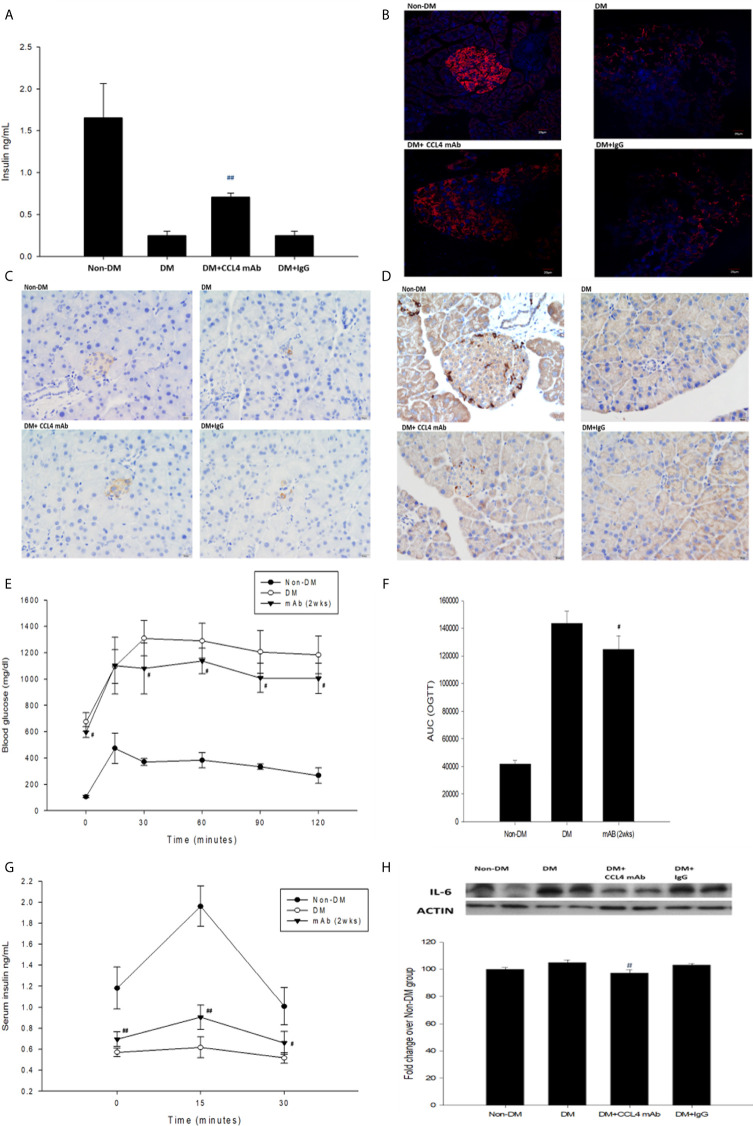
The effects of CCL4 inhibition on pancreatic tissues from STZ-induced diabetic mice. Serum insulin levels in STZ-induced diabetic mice (n=9; **A**). The CCL4 inhibition groups showed more insulin (red; **B**) (dark brown; **C**) and Ki-67 (dark brown; **D**) expression in the pancreas than the IgG-treated STZ-induced diabetic mice. The OGTT was conducted after CCL4 antibody injections for 2 weeks (n=6; **E**). AUCs of the OGTT calculated from the original graph (n=6; **F**). Insulin levels during the OGTT (n=6; **G**). Western blot and statistical analyses of IL-6 expression in pancreatic tissue from STZ-induced diabetic mice (n=3; **H**). N represents the pancreas from each individual that was used for 3 independent experiments ^#^ *P*<0.05, ^##^ *P<0.01* compared with DM+IgG mice **(A, H)** or untreated DM mice **(E–G)**.

Blood sugar levels were increased in the STZ-induced diabetic mice and in the mice that received the CCL4 antibody injection for 2 weeks. The blood sugar levels were controlled in the mice that received the CCL4 antibody injection for 4 weeks **(** **Table 1** **)**. Mice were fasted for 6 hours, and the serum concentrations of insulin were decreased in the STZ-induced diabetic mice compared with those in the non-DM mice. Insulin levels were increased in mice that received the CCL4 antibody injection for 4 weeks compared with those in IgG-treated diabetic mice **(** **Figure 1A** **)**. In type 1 DM, loss of islet β-cells requires therapeutic intervention to restore the β-cell mass or stimulate β-cell proliferation (16). In this study, fluorescence microscopy revealed that the pancreatic levels of insulin **(** **Figures 1B, C** **)** and the marker of proliferation Ki-67 expression **(** **Figure 1D** **)** were increased and the pancreatic morphology was more intact, complete, and regular with large pancreatic volume and closely arranged pancreatic β-cells in the CCL4 inhibition group than in the IgG-treated diabetic group. To assess glucose homeostasis in these mice, OGTTs and insulin levels during the tests were evaluated. The blood sugar levels were lower in the CCL4 inhibition group at 30, 60, 90 and 120 minutes than those in the untreated diabetic group **(** **Figure 1E** **)**. The AUCs of the OGTT were lower in the CCL4 antibody-treated group than those in the untreated diabetic group **(** **Figure 1F** **)**. The insulin concentrations in the CCL4 inhibition group were increased time 0 to 30 minutes after oral glucose loading **(** **Figure 1G** **)**. Taken together, the results from the OGTT implied that CCL4 inhibition could increase glucose clearance by increasing insulin secretion. These findings indicate the capability of inhibiting CCL4 to protect the pancreas in STZ-induced diabetic mice. Furthermore, the expression levels of IL-6 in pancreatic tissues were decreased in the CCL4 antibody injection group **(** **Figure 1H** **)**. Moreover, we used female NOD mice as another type 1 DM model. The results revealed that the blood sugar levels were decreased in the mice that received the CCL4 antibody injection for 4 weeks compared with those in the untreated DM mice **(** **Table 2** **)**.

**Table 1:** Blood sugar levels in STZ-induced diabetic mice.

Time point Groups	Pre- CCL4 mAb injection (mg/dl)	Post- CCL4 mAb injection (mg/dl)
Non-DM control	104.7 ± 13.1	108.2 ± 8.5
DM	335.7 ± 67.1	440.5 ± 24.8*
DM + CCL4 mAb for 2 wks + without mAb treatment for 2 wks	332.8 ± 55.4	392.7 ± 32.3
DM + CCL4 mAb for 4 wks	331.2 ± 42.9	259.8 ± 89.3(*)
DM + IgG_2A_	319.0 ± 24.4	404.0 ± 45.3*

Values are presented as the mean ± SD (n=4 in IgG_2A_-treated group; n=6~8 in the other groups). *P<0.05 and (*)P=0.051 compared with the same group of blood sugar levels before CCL4 mAb injection.

**Table 2:** Blood sugar levels in NOD mice.

Time points Groups	Pre- treatment (12-week-old, mg/dl)	Post- CCL4 mAb injection for 2 wks (14-week-old, mg/dl)	Post- CCL4 mAb injection for 4 wks (16-week-old, mg/dl)
NOD mice without treatment	73.2 ± 10.1	97.4 ± 10.2	150.4 ± 7.8
NOD mice + CCL4 mAb treatment	73.7 ± 8.9	85.7 ± 14.2	119.7 ± 23.4*

Values are presented as the mean ± SD (n=5 in the NOD control group; n=6~8 in the other group). *P<0.05 compared with the same group of blood sugar levels before CCL4 mAb injection.

### CCL4 Inhibition Protected Pancreatic β-Cells Under STZ Treatment *In Vitro*

**Figure 2:**
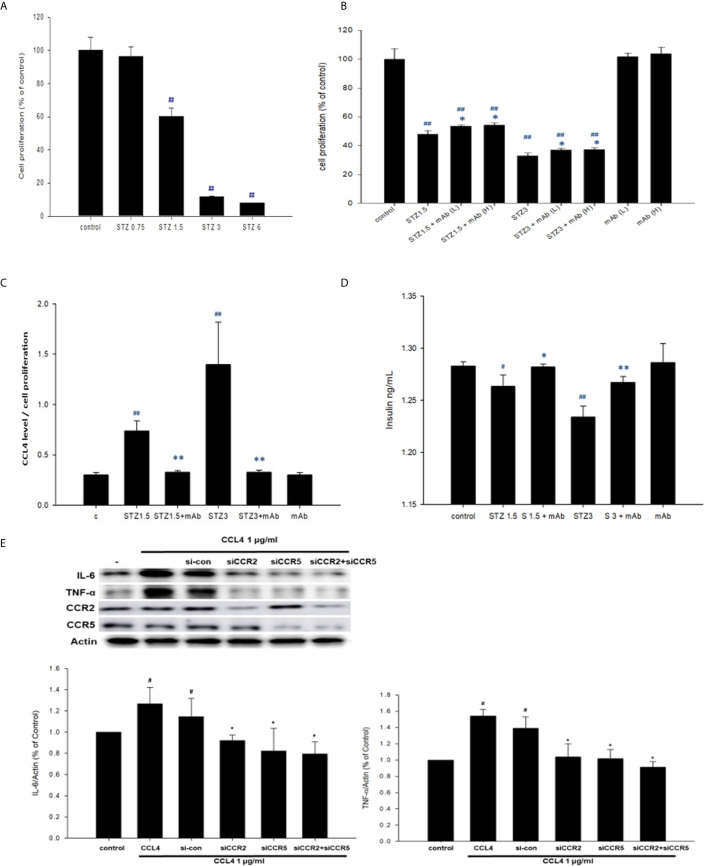
The effects of CCL4 on inflammation modulation in pancreatic β-cells. NIT-1 cell proliferation after STZ treatments at different doses (n=3; **A**). STZ-treated NIT-1 cells were then incubated with the CCL4 mAb at a low dose (L) and high dose (H). NIT-1 cell proliferation was evaluated using an MTT assay (n=3; **B**). The CCL4 levels in the supernatants of NIT-1 cells were standardized by the average MTT assay data (n=6; **C**). Insulin levels in the supernatants of NIT-1 cells (n=6; **D**). Western blot and statistical analyses of IL-6 and TNF-α expression in NIT-1 cells (n=3; **E**). ^#^ *P<0.05*, ^##^ *P<0.01* compared with the control group of NIT-1 cells. *P<0.05, **P<0.01 compared with the same STZ concentration-treated or CCL4-treated NIT-1 cell group.

In the *in vitro* experiment, we added STZ to mimic the *in vivo* conditions in our type 1 DM model. NIT-1 cell viability was decreased after STZ treatment **(** **Figure 2A** **)**. Interestingly, both relatively low and high doses of the CCL4 inhibitor enhanced NIT-1 cell proliferation after STZ stimulation **(** **Figure 2** **B)**. Because of the toxicity of STZ, we standardized the CCL4 levels in the NIT-1 cell supernatant. The standardized data showed that CCL4 could be induced by STZ stimulation and that this enhanced CCL4 level could be neutralized by the CCL4 antibody **(** **Figure 2C** **)**. Furthermore, insulin expression in the NIT-1 cell supernatants was decreased in the STZ-treated groups but reversed after CCL4 antibody treatment **(** **Figure 2D** **).** The above *in vitro* data suggested the direct protection provided by CCL4 inhibition on pancreatic β-cells, which was consistent with the *in vivo* findings.

### CCL4 Caused Pancreatic β-Cell Inflammation Through CCR2 and CCR5 *In Vitro*

Moreover, CCL4 treatments could induce IL-6 and TNF-α expressions in NIT-1 cells. The induced expressions of the above inflammatory proteins could be reversed by siRNA of CC chemokine receptor (CCR) 2 and CCR5. And, co-treatment with CCR2 and CCR5 did not further decrease CCL4-induced IL-6 and TNF-α expressions in NIT-1 cells **(** **Figure 2E** **)**. These results indicated that CCL4 could promote inflammation in pancreatic β-cells by different receptors.

### CCL4 Inhibition Protected Pancreatic Islet Cell Function and Decreased Insulin Resistance in db/db Mice

**Figure 3:**
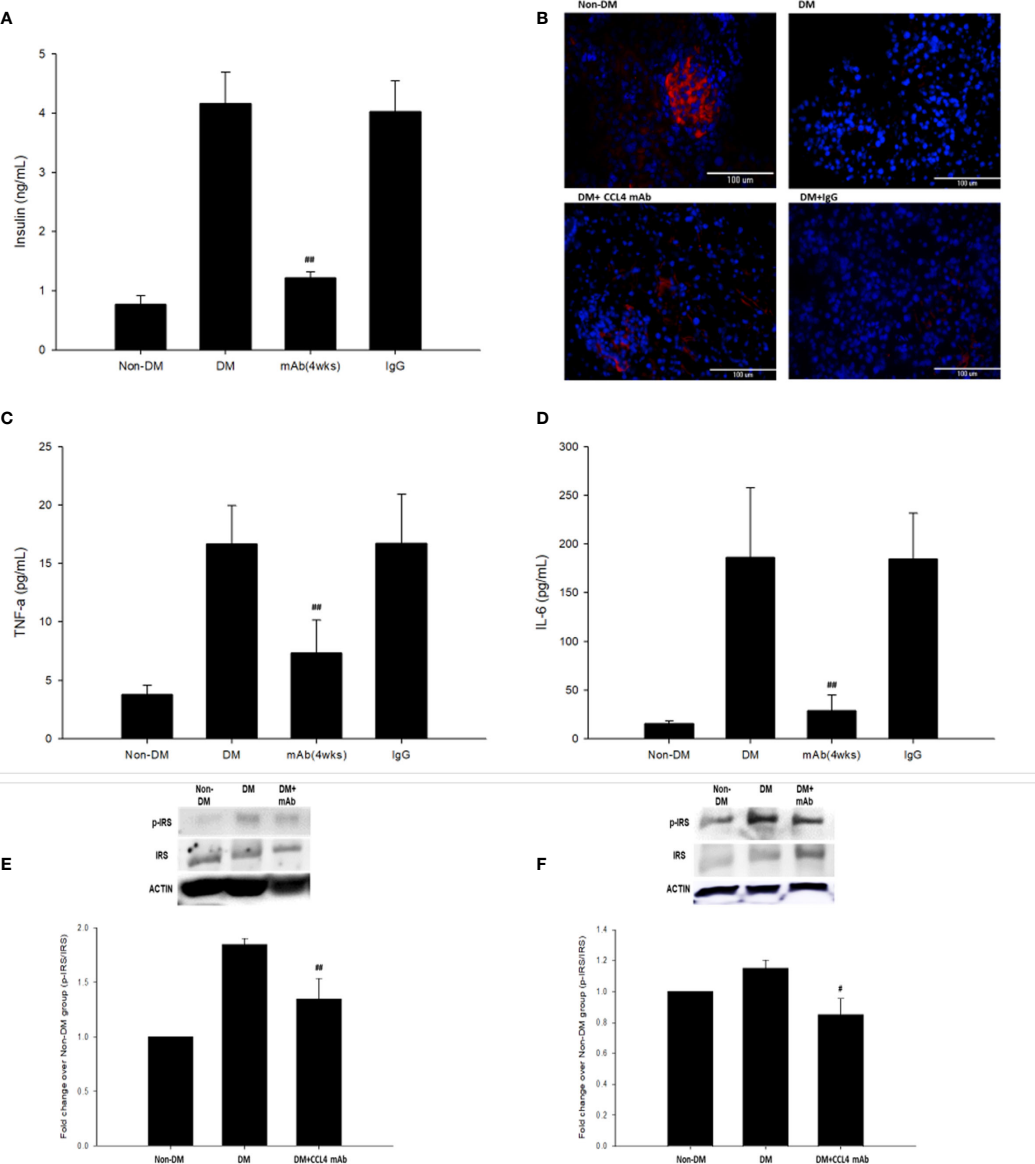
The effects of CCL4 inhibition on pancreatic tissues from db/db mice. Serum insulin levels in db/db mice (n=6; **A**). The CCL4 inhibition groups showed more insulin expression (red) in the pancreas than the IgG-treated diabetic db/db mice **(B)**. Serum TNF-α and IL-6 levels in db/db mice (n=6; **C, D**). CCL4 inhibition prevented the increased phosphorylation of IRS-1 in skeletal muscle and liver tissues (n=3; **E, F**) indicating the improvement of insulin signaling in db/db mice. ^#^ *P*<0.05, ^##^ *P<0.01* compared with DM+IgG mice **(A, C, D)** or untreated DM mice **(E, F)**.

Blood sugar concentrations were measured during experimental periods. The blood sugar levels of nontreated db/db mice were increased compared with those in the non-DM mice. The blood sugar levels in mice treated with a CCL4 antibody injection for 2 weeks were higher than those in the non-DM control group mice but relatively lower than those in the IgG-treated db/db mice. Interestingly, blood sugar levels were controlled in the group of mice that received the CCL4 antibody injection for 4 weeks **(** **Table 3** **)**. Compared with those in IgG-treated db/db mice, the serum insulin levels in the db/db mice receiving the CCL4 antibody treatment were reduced (**Figure 3A**). The insulin-positive areas per islet cell were increased in pancreatic islets from the CCL4 antibody-treated db/db mice compared to those in pancreatic islets from IgG-treated db/db mice. The pancreatic morphology was more intact with large pancreatic volume and closely arranged pancreatic β-cells in the CCL4 inhibition group than in the IgG-treated db/db mice **(** **Figure 3B** **)**. We performed the OGTT experiments using db/db mice. However, the blood sugar levels did not decline during the observation period, which might have been due to the high blood sugar levels in db/db mice at 12 weeks of age. In DM mice, the CCL4 inhibition group had lower levels of HOMA-IR than the IgG control group (CCL4 inhibition for 4 weeks group (32.76 ± 5.37) vs. DM+IgG group (180.28 ± 30.42), *P<0.01*). The levels of inflammatory markers, such as TNF-α and IL-6, which are the root causes of insulin resistance in type 2 diabetic patients with type 2 diabetes, were decreased in the CCL4 antibody injection group, compared with those in the IgG-treated DM group **(** **Figure 3D** **)**. Insulin resistance to glucose transport and metabolism leads to the development of type 2 diabetes. The enhanced IRS-1 serine307 phosphorylation and defective IRS-1-dependent signaling results in reduced glucose transport activity (17–19). We also analyzed the changes in proteins related to insulin resistance, resulting in the inhibition of insulin signaling. CCL4 antibody-treated mice had decreased levels of phosphorylated IRS-1 in both skeletal muscle and liver tissues compared to those in the untreated db/db mice **(** **Figures 3E, F** **)**, implying the beneficial effects of CCL4 inhibition on improving insulin resistance.

**Table 3:** Blood sugar levels in db/db mice.

Time pointsGroups	Pre- CCL4 mAb injection(8-week-old, mg/dl)	Post- CCL4 mAb injection(12-week-old, mg/dl)
Non-DM control (db/m mice)	134.0 ± 55.1	135.8 ± 57.4
DM (db/db mice)	325.3 ± 57.0	656.3 ± 39.7**
DM + CCL4 mAb for 2 wks + without mAb treatment for 2 wks	321.0 ± 55.1	473.7 ± 58.5**
DM + CCL4 mAb for 4 wks	342.7 ± 58.0	377.0 ± 33.6
DM + IgG_2A_	363.3 ± 82.2	629.8 ± 75.5**

Values are presented as the mean ± SD (n=4 in IgG_2A_-treated group; n=5~8 in the other groups). **P<0.01 compared with the same group of blood sugar levels before CCL4 mAb injection.

### CCL4 Inhibition Protected Pancreatic Islet Function and Decreased Insulin Resistance in Mice Fed With a High-Fat Diet

**Figure 4:**
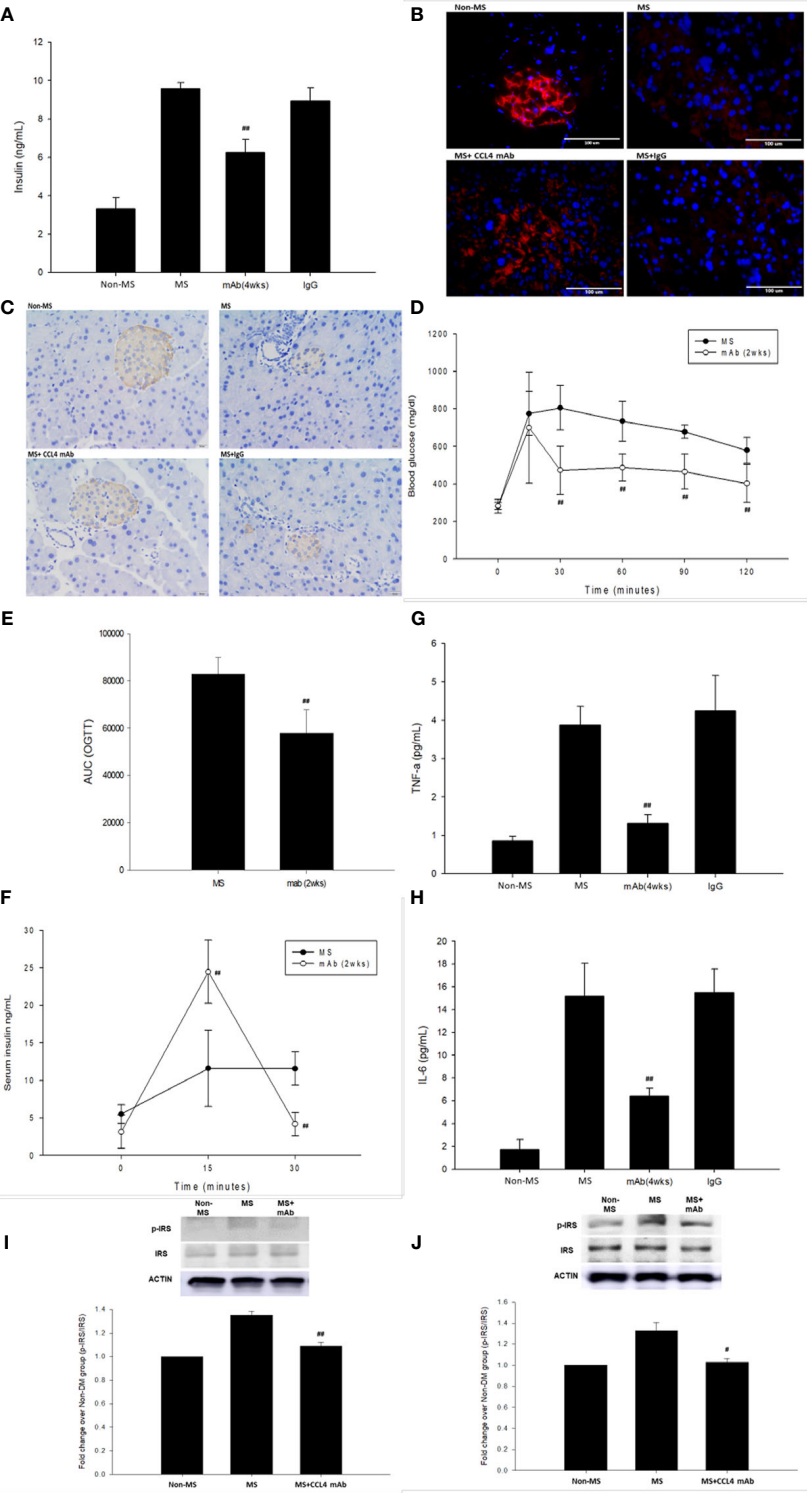
The effects of CCL4 inhibition on pancreatic tissues from metabolic syndrome mice. Serum insulin levels in high-fat diet metabolic syndrome mice (n=6; **A**). The CCL4 inhibition groups showed more insulin expression in the pancreas than the IgG-treated metabolic syndrome mice (red; **B**) (dark brown; **C**). The OGTT was conducted after CCL4 antibody injections for 2 weeks (n=6; **D**). AUCs of the OGTT calculated from the original graph (n=6; **E**). Insulin levels during the OGTT (n=6; **F**). Serum TNF-α and IL-6 levels in metabolic syndrome mice (n=6; **G, H**). CCL4 inhibition prevented the increased phosphorylation of IRS-1 in skeletal muscle and liver tissues (n=3; **I, J**), indicating the improvement of insulin signaling in metabolic syndrome mice. ^#^>*P*<0.05, ^##^ *P<0.01* compared with DM+IgG mice **(A, G, H)** or untreated metabolic syndrome mice **(D–F, I, J)**.

C57BL/6 mice were fed a high-fat diet to mimic the clinical development of metabolic syndrome. This group of mice was defined as the metabolic syndrome model. The blood sugar levels were increased in metabolic syndrome mice (fed a high-fat diet) compared to those in nonmetabolic syndrome mice (fed a normal diet). Blood sugar levels were decreased in the groups that received the CCL4 antibody injection for 4 weeks **(** **Table 4** **)**. In addition, mice receiving the CCL4 antibody showed lower serum insulin levels (**Figure 4A**), higher insulin-positive areas and more intact with large pancreatic volume and closely arranged pancreatic β-cells morphology **(** **Figures 4B, C** **)** than the IgG-treated metabolic syndrome mice. The OGTTs and insulin levels during the tests were evaluated. The blood sugar levels were lower in the CCL4 antibody-treated group at 30, 60, 90 and 120 minutes than in the untreated metabolic syndrome group **(** **Figure 4D**). The AUCs of the OGTT were lower in the CCL4 inhibition group **(** **Figure 4E** **)**. The insulin concentrations in the CCL4 inhibition group were increased at 15 minutes but were significantly lower at 30 minutes compared to those in the untreated metabolic syndrome group during the OGTT **(** **Figure 4F)**. Taken together, data from the OGTT revealed that CCL4 inhibition could increase glucose clearance and insulin sensitivity. In metabolic syndrome mice, the CCL4 inhibition group had lower levels of HOMA-IR than the IgG control group (CCL4 inhibition for 4 weeks group (89.33 ± 11.41) vs. DM+IgG group (161.17 ± 14.52), *P<0.01*). Furthermore, the TNF-α and IL-6 levels were decreased in the CCL4 antibody-treated group compared with those in the IgG-treated metabolic syndrome group **(** **Figures 4G, H** **)**. CCL4 inhibition mice also had decreased levels of phosphorylated IRS-1 in both skeletal muscle and liver tissues compared to those in the untreated metabolic syndrome mice **(** **Figures 4I, J** **)**, implying the beneficial effects of CCL4 inhibition on insulin signaling. The above data imply the beneficial effects of CCL4 inhibition on systemic inflammation, pancreatic islet cell function and insulin resistance in metabolic syndrome mice.

**Table 4:** Blood sugar levels in C57BL/6 mice fed a high-fat diet.

Time pointsGroups	Pre- CCL4 mAb injection (mg/dl)	Post- CCL4 mAb injection (mg/dl)
Normal diet control	107.8 ± 14.0	108.7 ± 8.1
Metabolic syndrome	233.2 ± 22.6	245.4 ± 18.7
Metabolic syndrome + CCL4 mAb for 2 wks + without mAb treatment for 2 wks	245.5 ± 65.4	208.0 ± 11.4
Metabolic syndrome + CCL4 mAb for 4 wks	243.9 ± 27.3	208.6 ± 24.4**
Metabolic syndrome + IgG_2A_	233.5 ± 14.9	252.8 ± 4.9

This group was defined as metabolic syndrome mice. Values are presented as the mean ± SD (n=4 in IgG_2A_-treated group; n=6~13 in the other groups). **P<0.01 compared with the same group of blood sugar levels before CCL4 mAb injection.

## Discussion

There are several main findings in this study. First, blood sugar levels were increased in the STZ-induced diabetic mice (a model simulating type 1 DM), which were maintained by treatment with a CCL4 antibody. Furthermore, inhibition of CCL4 with an antibody decreased pancreatic IL-6 expression, increased β-cell proliferation, restored the β-cell mass, and increased pancreatic and circulating insulin levels in the STZ-induced diabetic mouse model. Additionally, *in vitro* treatment with a CCL4 antibody protected pancreatic β-cells from STZ stimulation, suggesting that the autocrine and/or paracrine effects of CCL4 can be directly inhibited by an antibody. Interestingly, direct administration of CCL4 resulted in enhanced inflammatory protein expressions such as IL-6 and TNF-α mainly *via* the CCR2 signaling pathway in pancreatic β-cells. Second, treatment with the CCL4 antibody could retard the elevation of blood sugar levels in db/db mice (a model simulating type 2 DM) and reduce the blood sugar levels in mice fed a high fat diet (a model simulating metabolic syndrome). In both the type 2 diabetes and metabolic syndrome models, treatment with the CCL4 antibody increased pancreatic insulin expression, and reduced circulating insulin, TNF-α, and IL-6 levels. It also increased the glucose clearance ability and insulin sensitivity and modulated the insulin resistance signaling pathway with decreased phosphorylation of IRS-1 in both skeletal muscle and liver tissues. Taken together, these results suggest that while reducing local inflammation and protecting pancreatic β cells *via* increased insulin expression in a type 1 DM animal model, inhibition of CCL4 with a specific antibody could also attenuate systemic inflammation, improve insulin resistance, and reduce circulating insulin levels in both type 2 diabetes animal models and metabolic syndrome animal models. The findings suggest the novel and common roles of CCL4-related inflammation in the progression of hyperglycemia in different experimental DM.

DM is suggested to be a metabolic disease related to inflammation and vascular complications resulting from reduced insulin production or a decreased tissue response to insulin (20, 21). Insulin resistance is a pathogenic feature of type 2 diabetes that contributes to cardiovascular diseases (22). CCL4 has the ability to attract macrophages, and these macrophages are implicated in the destruction of islet cells (23, 24). In islet cells, CD40-CD40L interactions induce the secretion of CCL4 and contribute to β-cell death and early islet graft loss (25). The circulating concentrations of CCL4 do not differ between patients with type 1 and type 2 diabetes, suggesting a general involvement of CCL4 in different types of diabetes (9). Insulin infusion could suppress the CCL4 expression in mononuclear cells from patients with type 2 diabetes (26). Circulating CCL4 levels are also reduced in association with decreased β-cell stress, as shown by a reversed association with proinsulin (8). In the current study, we demonstrated that CCL4 may be related to local inflammation *in vitro* as well as to systemic inflammation *in vivo*. While there are no previous articles mentioning about the production of CCL4 from NIT-1 cells (11), we observed for the first time that the pancreatic β-cell line NIT-1 could release CCL4 after STZ stimulation. More importantly, direct blockade of CCL4 could reverse pancreatic islet cell damage and/or systemic insulin resistance to stabilize the blood sugar levels in both the type 1 and type 2 diabetes models and in the metabolic syndrome animal model. Our findings provide both *in vitro* and *in vivo* evidence to the novel and common role of CCL4 for blood sugar deterioration in different types of diabetic animal models. It might be particularly important in clinical type 2 DM since a significant portion of the patients may finally require insulin supplement due to pancreatic islet failure despite the use of oral glucose lowering agents.

Phosphorylation of IRS-1 at Ser307, a potential mechanism underlying insulin resistance, attenuates insulin signaling pathways (19). It has been shown that targeting anti-inflammatory cytokines might restore insulin sensitivity (27, 28). Furthermore, diabetic drugs with anti-inflammatory properties protect against cardiovascular complications in patients with type 2 diabetes (29). On the other hand, anti-hypertensive drugs were shown to enhance insulin sensitivity (30, 31), implying that improving the cardiovascular system might exert beneficial effects on insulin resistance. In this study, we revealed for the first time that direct inhibition of CCL4 could reverse the impaired insulin signaling pathway by decreasing the phosphorylation of IRS-1 in skeletal muscle and liver tissues in mice with both type 2 DM and metabolic syndrome. These results suggested the direct beneficial effects of CCL4 inhibition on *in vivo* insulin resistance. Furthermore, systemic inflammation, indicated by serum TNF-α and IL-6 levels, was also decreased by CCL4 inhibition in type 2 DM and metabolic syndrome mouse models in this study. It has been indicated that inflammatory mediators such as TNF-α and IL-6 are increased with insulin resistance and play an important role in deregulating glucose homeostasis in type 2 DM (32). However, recent clinical studies failed to demonstrate the beneficial effect of TNF-α neutralization on insulin resistance, insulin sensitivity, and endothelial function in patients with type 2 diabetes (33, 34). While both TNF-α and IL-6 could be reduced by CCL4 inhibition, it will be interesting to elucidate whether a broader inhibition of systemic inflammation with a specific chemokine, such as CCL4, could improve insulin resistance in clinical diabetes.

In this study, CCL4 inhibition was shown to ameliorate inflammatory proteins in different DM models. The CCL4-mediated signal pathways should be further clarified. CCR5, the most well-known receptor of CCL4, is linked with obesity, inflammation, and insulin resistance (35, 36). Nevertheless, transient blockade of CCR5 or CCR5 deficiency accelerated rather than prevented type 1 diabetes in NOD mice (37, 38). CCL4 can also signal through CCR2 (39–41). Targeting of CCR2 may lead to therapies against type 1 DM in NOD mice. Dual blockade of CCR2 and CCR5 was thought to be a potential therapeutic approach for immunologic and cardiovascular diseases (42). In addition, dual CCR2/CCR5 antagonism could ameliorate insulin resistance and inflammation in high-fat diet-fed mice and decrease CCL2/CCL4‐induced migration of macrophage (43). However, clinical study of a dual chemokine CCR2/5 receptor antagonist related to the albuminuria in adults with diabetic nephropathy was discontinued despite the safety profile was shown (44). One of the possibility is that both CCR2 and CCR5 are responsible for multiple ligands (45). Given the unpredictable off-target effects, blockade of CCR2 and/or CCR5 might not the best option for therapy. Moreover, based on the CCL3/CCL4 and their NH2-terminal processing, several CCRs inclusive of CCR1, CCR2, CCR3 and CCR5 are recognized (46). As the result, further investigations are required to clarify the main receptor participating in the downstream inflammatory pathways and contributes to the beneficial effects of CCL4 inhibition in DM. In our study, the CCL4-induced inflammatory protein expressions could be abolished mainly by siRNA of CCR2 and only partially by siRNA of CCR5, suggesting both CCR2 and CCR5 may contribute to the effects of CCL4 on pancreatic β-cells. However, given the unsuccessful results in previous clinical trials with dual CCR2/5 receptor antagonist, the direct inhibition of CCL4 itself might be another potential strategy rather than targeting its receptors.

There are some limitations that should be mentioned in the current study. First, it has been previously indicated that CCL4 could be produced by neutrophils, monocytes, B cells, T cells, fibroblasts, endothelial cells, and epithelial cells *in vivo* (47, 48). We recently showed that either TNF-a or oxidized low-density lipoprotein could directly induce CCL4 expression in human coronary artery endothelial cells and macrophages (39). In the current study, the particular types of cells expressing CCL4 may be identified to explore the potential targets of CCL4 antibody in individual models of animals. However, given the general effects of CCL4 antibody on blood sugar in different animal models, one may speculate that the main target of CCL4 *in vivo* may be circulating CCL4 rather than individual types of cells. It should be further investigated in future studies. Also, in the current study, while the *in vitro* study showed a direct protective effect of CCL4 inhibition on pancreatic islet cells, it is possible that CCL4 might be also induced in pancreatic islet cells *in vivo*. Further experiments are required to elucidate the molecular mechanisms in detail. Second, while CCL4 inhibition could improve the phosphorylation of IRS-1 in skeletal muscle and liver tissues in type 2 DM and metabolic syndrome model mice, further experiments are needed to explore the role of CCL4-related inflammation in the development of insulin resistance, such as inflammation in adipose tissues. Interestingly, the level of serum IL-6 was obviously higher in the type 2 diabetic model but the level of serum TNF-α was relatively similar among different animal models. One speculation might be that the molecular signaling pathways are different between IL-6 and TNF-α and their sources of secretion are different. The accumulation of obese adipose tissue in the dbdb mice might lead to starkly enhanced level of IL-6 (49). In this study, we focused on the effects of CCL4 inhibition on the levels of TNF-α and IL-6, which may be related to the root causes of insulin resistance in type 2 diabetes. However, some other mechanisms involving the systemic insulin resistance and pancreatic inflammation such as IL-10 and so on should also be critical. Accordingly, a complete pattern of serum pro- and anti-inflammatory cytokines and an analysis of the inflammatory cell infiltration in the pancreas should be measured to fully understand the role of CCL4 in our future studies. Third, in the current study, mice were repeatedly injected by the antibody of different species. It may be necessary to consider the effects of the injected antibody on mice especially the impact of anti-CCL4 neutralizing antibody on immunophenotype of these mice during the experiment periods. Fourth, this study is limited to mouse models and hence the data should be interpreted in the light of those limiting factors. Further human studies are needed to validate the results. And, the binding affinity, specificity, and pharmacodynamics of the CCL4-specific antibody should be investigated before its application in the clinical treatment of DM. Accordingly, the current findings may serve as pilot proof-of-concept data. Additional experiments are required to determine the dose and time-dependent effects of CCL4-specific antibodies. If they exist, other substances or chemicals that may modify the effects of CCL4 should also be tested to validate and elucidate the role of CCL4-related inflammatory mechanisms in other animal models, especially in large animal models.

In conclusion, systemic and/or direct inhibition of CCL4 protected pancreatic islet cells, improved insulin resistance, and retarded the progression of hyperglycemia in different experimental DM models. Our findings not only elucidate the potential role of CCL4-related inflammation in the progression of hyperglycemia but also provide some novel mechanistic clues to the deterioration of blood sugar levels in the presence of DM. It may be particularly important in type 2 DM since a significant portion of the patients may progress to pancreatic islet failure and rely on insulin therapy despite the use of multiple oral glucose lowering agents. Future studies may be performed to evaluate whether CCL4 could be a potential therapeutic target, with either monoclonal antibody or small molecule drugs, to protect pancreatic islet and stabilize blood sugar in clinical DM.

## Data Availability Statement

The original contributions presented in the study are included in the article/supplementary material. Further inquiries can be directed to the corresponding author.

## Ethics Statement

The animal study was reviewed and approved by the Institutional Animal Care and Use Committee (IACUC) protocol approved by National Yang-Ming University (IACUC no. 1050909).

## Author Contributions

T-TC was the main conductor of this study and contributed to the study conception and design, implementation, statistical analysis, interpretation, and the preparation of the manuscript. L-YL revised the manuscript. J-WC supervised the study conduction and contributed to the study conception and design, implementation, statistical interpretation, the preparation and finalization of the manuscript. All authors contributed to the article and approved the submitted version.

## Funding

This work was supported by the Taipei Veterans General Hospital, Taipei, Taiwan [V105E18-004-MY3, V105C-117, and V104C-101].

## Conflict of Interest

The authors declare that the research was conducted in the absence of any commercial or financial relationships that could be construed as a potential conflict of interest.

## References

[1] Akash MS Rehman K Chen S. Role of Inflammatory Mechanisms in Pathogenesis of Type 2 Diabetes Mellitus. J Cell Biochem (2013) 114(3):525–31. 10.1002/jcb.24402 22991242

[2] Abdel-Moneim A Bakery HH Allam G. The Potential Pathogenic Role of IL-17/Th17 Cells in Both Type 1 and Type 2 Diabetes Mellitus. Biomed Pharmacother (2018) 101:287–92. 10.1016/j.biopha.2018.02.103 29499402

[3] Deng F Wang S Zhang L. Endothelial Microparticles Act as Novel Diagnostic and Therapeutic Biomarkers of Diabetes and Its Complications: A Literature Review. BioMed Res Int (2016) 2016:9802026. 10.1155/2016/9802026 27803933 PMC5075589

[4] Chawla A Chawla R Jaggi S. Microvasular and Macrovascular Complications in Diabetes Mellitus: Distinct or Continuum? Indian J Endocrinol Metab (2016) 20(4):546–51. 10.4103/2230-8210.183480 PMC4911847 27366724

[5] Bao X Borne Y Johnson L Muhammad IF Persson M Niu K. Comparing the Inflammatory Profiles for Incidence of Diabetes Mellitus and Cardiovascular Diseases: A Prospective Study Exploring the ‘Common Soil’ Hypothesis. Cardiovasc Diabetol (2018) 17(1):87. 10.1186/s12933-018-0733-9 29895294 PMC5996509

[6] Noels H Weber C Koenen RR. Chemokines as Therapeutic Targets in Cardiovascular Disease. Arterioscler Thromb Vasc Biol (2019) 39(4):583–92. 10.1161/atvbaha.118.312037 30760014

[7] Hanifi-Moghaddam P Kappler S Seissler J Muller-Scholze S Martin S Roep BO. Altered Chemokine Levels in Individuals At Risk of Type 1 Diabetes Mellitus. Diabetes Med (2006) 23(2):156–63. 10.1111/j.1464-5491.2005.01743.x 16433713

[8] Pfleger C Kaas A Hansen L Alizadeh B Hougaard P Holl R. Relation of Circulating Concentrations of Chemokine Receptor CCR5 Ligands to C-peptide, Proinsulin and HbA1c and Disease Progression in Type 1 Diabetes. Clin Immunol (2008) 128(1):57–65. 10.1016/j.clim.2008.03.458 18434252

[9] Pham MN Hawa MI Roden M Schernthaner G Pozzilli P Buzzetti R. Increased Serum Concentrations of Adhesion Molecules But Not of Chemokines in Patients With Type 2 Diabetes Compared With Patients With Type 1 Diabetes and Latent Autoimmune Diabetes in Adult Age: Action LADA 5. Diabetes Med (2012) 29(4):470–8. 10.1111/j.1464-5491.2011.03546.x 22150724

[10] Chang TT Lin LY Chen JW. Inhibition of Macrophage Inflammatory Protein-1β Improves Endothelial Progenitor Cell Function and Ischemia-Induced Angiogenesis in Diabetes. Angiogenesis (2019) 22(1):53–65. 10.1007/s10456-018-9636-3 29987448

[11] Chang TT Chen JW. Emerging Role of Chemokine CC Motif Ligand 4 Related Mechanisms in Diabetes Mellitus and Cardiovascular Disease: Friends or Foes? Cardiovasc Diabetol (2016) 15(1):117. 10.1186/s12933-016-0439-9 27553774 PMC4995753

[12] Makino S Kunimoto K Muraoka Y Mizushima Y Katagiri K Tochino Y. Breeding of a Non-Obese, Diabetic Strain of Mice. Jikken Dobutsu (1980) 29(1):1–13. 10.1538/expanim1978.29.1_1 6995140

[13] Pamidi S Wroblewski K Broussard J Day A Hanlon EC Abraham V. Obstructive Sleep Apnea in Young Lean Men: Impact on Insulin Sensitivity and Secretion. Diabetes Care (2012) 35(11):2384–9. 10.2337/dc12-0841 PMC3476882 22912423

[14] Maugham ML Thomas PB Crisp GJ Philp LK Shah ET Herington AC. Insights From Engraftable Immunodeficient Mouse Models of Hyperinsulinaemia. Sci Rep (2017) 7(1):491. 10.1038/s41598-017-00443-x 28352127 PMC5428450

[15] Friedewald WT Levy RI Fredrickson DS. Estimation of the Concentration of Low-Density Lipoprotein Cholesterol in Plasma, Without Use of the Preparative Ultracentrifuge. Clin Chem (1972) 18(6):499–502. 10.1093/clinchem/18.6.499 4337382

[16] Zhou Q Melton DA. Pancreas Regeneration. Nature (2018) 557(7705):351–8. 10.1038/s41586-018-0088-0 PMC6168194 29769672

[17] Meagher C Arreaza G Peters A Strathdee CA Gilbert PA Mi QS. CCL4 Protects From Type 1 Diabetes by Altering Islet Beta-Cell-Targeted Inflammatory Responses. Diabetes (2007) 56(3):809–17. 10.2337/db06-0619 17327452

[18] Nakamori Y Emoto M Fukuda N Taguchi A Okuya S Tajiri M. Myosin Motor Myo1c and its Receptor NEMO/IKK-gamma Promote TNF-alpha-Induced serine307 Phosphorylation of IRS-1. J Cell Biol (2006) 173(5):665–71. 10.1083/jcb.200601065 PMC2063884 16754954

[19] Hossain Z Valicherla GR Gupta AP Syed AA Riyazuddin M Chandra S. Discovery of Pancreastatin Inhibitor PSTi8 for the Treatment of Insulin Resistance and Diabetes: Studies in Rodent Models of Diabetes Mellitus. Sci Rep (2018) 8(1):8715. 10.1038/s41598-018-27018-8 29880906 PMC5992141

[20] Domingueti CP Dusse LM Carvalho M de Sousa LP Gomes KB Fernandes AP. Diabetes Mellitus: The Linkage Between Oxidative Stress, Inflammation, Hypercoagulability and Vascular Complications. J Diabetes Complications (2016) 30(4):738–45. 10.1016/j.jdiacomp.2015.12.018 26781070

[21] Derosa G Maffioli P. A Review About Biomarkers for the Investigation of Vascular Function and Impairment in Diabetes Mellitus. Vasc Health Risk Manag (2016) 12:415–9. 10.2147/vhrm.s64460 PMC5108560 27877049

[22] Henry RR. Insulin Resistance: From Predisposing Factor to Therapeutic Target in Type 2 Diabetes. Clin Ther (2003) 25 Suppl B:B47–63. 10.1016/S0149-2918(03)80242-4 14553866

[23] DeVries ME Ran L Kelvin DJ. On the Edge: The Physiological and Pathophysiological Role of Chemokines During Inflammatory and Immunological Responses. Semin Immunol (1999) 11(2):95–104. 10.1006/smim.1999.0165 10329496

[24] Benoist C Mathis D. Cell Death Mediators in Autoimmune Diabetes–No Shortage of Suspects. Cell (1997) 89(1):1–3. 10.1016/S0092-8674(00)80174-9 9094706

[25] Barbe-Tuana FM Klein D Ichii H Berman DM Coffey L Kenyon NS. CD40-CD40 Ligand Interaction Activates Proinflammatory Pathways in Pancreatic Islets. Diabetes (2006) 55(9):2437–45. 10.2337/db05-1673 16936191

[26] Ghanim H Korzeniewski K Sia CL Abuaysheh S Lohano T Chaudhuri A. Suppressive Effect of Insulin Infusion on Chemokines and Chemokine Receptors. Diabetes Care (2010) 33(5):1103–8. 10.2337/dc09-2193 PMC2858184 20200310

[27] Johnson AMF Hou S Li P. Inflammation and Insulin Resistance: New Targets Encourage New Thinking: Galectin-3 and LTB4 are Pro-Inflammatory Molecules That can be Targeted to Restore Insulin Sensitivity. Bioessays (2017) 39(9):10. 10.1002/bies.201700036 PMC5709518 28752547

[28] Luna-Vital D Weiss M Gonzalez de Mejia E. Anthocyanins From Purple Corn Ameliorated Tumor Necrosis Factor-Alpha-Induced Inflammation and Insulin Resistance in 3T3-L1 Adipocytes Via Activation of Insulin Signaling and Enhanced GLUT4 Translocation. Mol Nutr Food Res (2017) 61(12). 10.1002/mnfr.201700362 28759152

[29] Yaribeygi H Atkin SL Pirro M Sahebkar A. A Review of the Anti-Inflammatory Properties of Antidiabetic Agents Providing Protective Effects Against Vascular Complications in Diabetes. J Cell Physiol (2018) 234(6):8286–94. 10.1002/jcp.27699 30417367

[30] Jeon EJ Kim DY Lee NH Choi HE Cheon HG. Telmisartan Induces Browning of Fully Differentiated White Adipocytes Via M2 Macrophage Polarization. Sci Rep (2019) 9(1):1236. 10.1038/s41598-018-38399-1 30718686 PMC6362091

[31] Yanagihara H Ushijima K Arakawa Y Aizawa K Fujimura A. Effects of Telmisartan and Olmesartan on Insulin Sensitivity and Renal Function in Spontaneously Hypertensive Rats Fed a High Fat Diet. J Pharmacol Sci (2016) 131(3):190–7. 10.1016/j.jphs.2016.06.003 27430988

[32] De Felice FG Ferreira ST. Inflammation, Defective Insulin Signaling, and Mitochondrial Dysfunction as Common Molecular Denominators Connecting Type 2 Diabetes to Alzheimer Disease. Diabetes (2014) 63(7):2262–72. 10.2337/db13-1954 24931033

[33] Wascher TC Lindeman JH Sourij H Kooistra T Pacini G Roden M. Chronic TNF-alpha Neutralization Does Not Improve Insulin Resistance or Endothelial Function in “Healthy” Men With Metabolic Syndrome. Mol Med (Cambridge Mass) (2011) 17(3-4):189–93. 10.2119/molmed.2010.00221 PMC3060990 21103669

[34] Paquot N Castillo MJ Lefebvre PJ Scheen AJ. No Increased Insulin Sensitivity After a Single Intravenous Administration of a Recombinant Human Tumor Necrosis Factor Receptor: Fc Fusion Protein in Obese Insulin-Resistant Patients. J Clin Endocrinol Metab (2000) 85(3):1316–9. 10.1210/jcem.85.3.6417 10720082

[35] Ota T. CCR5: A Novel Player in the Adipose Tissue Inflammation and Insulin Resistance? Adipocyte (2013) 2(2):99–103. 10.4161/adip.22420 23805406 PMC3661115

[36] Kitade H Sawamoto K Nagashimada M Inoue H Yamamoto Y Sai Y. CCR5 Plays a Critical Role in Obesity-Induced Adipose Tissue Inflammation and Insulin Resistance by Regulating Both Macrophage Recruitment and M1/M2 Status. Diabetes (2012) 61(7):1680–90. 10.2337/db11-1506 PMC3379680 22474027

[37] Meagher C Beilke J Arreaza G Mi QS Chen W Salojin K. Neutralization of interleukin-16 Protects Nonobese Diabetic Mice From Autoimmune Type 1 Diabetes by a CCL4-dependent Mechanism. Diabetes (2010) 59(11):2862–71. 10.2337/db09-0131 PMC2963545 20693344

[38] Solomon M Balasa B Sarvetnick N. CCR2 and CCR5 Chemokine Receptors Differentially Influence the Development of Autoimmune Diabetes in the NOD Mouse. Autoimmunity (2010) 43(2):156–63. 10.3109/08916930903246464 19824873

[39] Chang TT Yang HY Chen C Chen JW. CCL4 Inhibition in Atherosclerosis: Effects on Plaque Stability, Endothelial Cell Adhesiveness, and Macrophages Activation. Int J Mol Sci (2020) 21(18):6567. 10.3390/ijms21186567 PMC7555143 32911750

[40] Guan E Wang J Norcross MA. Amino-Terminal Processing of MIP-1beta/CCL4 by CD26/dipeptidyl-peptidase IV. J Cell Biochem (2004) 92(1):53–64. 10.1002/jcb.20041 15095403

[41] Guan E Wang J Roderiquez G Norcross MA. Natural Truncation of the Chemokine MIP-1 Beta /CCL4 Affects Receptor Specificity But Not anti-HIV-1 Activity. J Biol Chem (2002) 277(35):32348–52. 10.1074/jbc.M203077200 12070155

[42] Zhao Q. Dual Targeting of CCR2 and CCR5: Therapeutic Potential for Immunologic and Cardiovascular Diseases. J Leukoc Biol (2010) 88(1):41–55. 10.1189/jlb.1009671 20360402

[43] Huh JH Kim HM Lee ES Kwon MH Lee BR Ko HJ. Dual CCR2/5 Antagonist Attenuates Obesity-Induced Insulin Resistance by Regulating Macrophage Recruitment and M1/M2 Status. Obesity (Silver Spring Md) (2018) 26(2):378–86. 10.1002/oby.22103 29280303

[44] Gale JD Gilbert S Blumenthal S Elliott T Pergola PE Goteti K. Effect of PF-04634817, an Oral CCR2/5 Chemokine Receptor Antagonist, on Albuminuria in Adults With Overt Diabetic Nephropathy. Kidney Int Rep (2018) 3(6):1316–27. 10.1016/j.ekir.2018.07.010 PMC6224665 30450458

[45] White GE Iqbal AJ Greaves DR. CC Chemokine Receptors and Chronic Inflammation–Therapeutic Opportunities and Pharmacological Challenges. Pharmacol Rev (2013) 65(1):47–89. 10.1124/pr.111.005074 23300131

[46] Menten P Wuyts A Van Damme J. Macrophage Inflammatory Protein-1. Cytokine Growth Factor Rev (2002) 13(6):455–81. 10.1016/S1359-6101(02)00045-X 12401480

[47] Morimoto T Takagi H Kondo T. Canine Pancreatic Allotransplantation With Duodenum (Pancreaticoduodenal Transplantation) Using Cyclosporin A. Nagoya J Med Sci (1985) 47(1-2):57–66. 3887178

[48] Morrison MD Lundquist PG. Labyrinthine Morphology and Temperature in Cryosurgery (Guinea Pig). Acta Otolaryngol (1974) 77(4):261–73. 10.3109/00016487409124624 4364832

[49] McArdle MA Finucane OM Connaughton RM McMorrow AM Roche HM. Mechanisms of Obesity-Induced Inflammation and Insulin Resistance: Insights Into the Emerging Role of Nutritional Strategies. Front Endocrinol (2013) 4:52. 10.3389/fendo.2013.00052 PMC3650620 23675368

